# The dynamics of psychological attributes and symptomatic comorbidity of depression in children and adolescents

**DOI:** 10.1007/s00127-023-02532-x

**Published:** 2023-07-21

**Authors:** Milagros Ocalin Sánchez Hernández, Francisco Pablo Holgado-Tello, Miguel Á. Carrasco

**Affiliations:** 1 National Autonomous University of Nicaragua, León (UNAN-León), León, Nicaragua; 2 National University of Distance Education (UNED), Madrid, Spain

**Keywords:** Network analysis, Comorbidity, Psychopathologies, Children, Adolescents

## Abstract

**Purpose:**

This investigation aimed to explore attribute dynamics and symptomatic comorbidity of depression with internalizing, externalizing, and other personal–contextual problems in children and adolescents from a network analysis.

**Methods:**

We tested an attribute network of regularized partial correlations, standard and alternative centrality measures, and comorbidity bridge symptoms according to centrality bridge measures.

**Results:**

Regularized partial correlation network and a centrality measures graph shown the prominent position of social problems and anxiety–depression. Minimum spanning tree (MST) found a hierarchical dynamics between attributes where mixed anxiety–depression was identified as the core and the other attributes were hierarchically connected to it by being positioned in six branches that are differentiated according to their theoretical contents. The most central connections are established with the attributes of their own community or theoretical groups, and 37 bridge symptoms were identified in all networks.

**Conclusions:**

A significant role of mixed anxiety depression as an activator and intermediary of psychopathologies was supported as a central attribute of internalizing problems. Aggressive behavior as part of the broad externalizing dimension was one of the constructs that most intensively activate the network, and social problems were also distinguished as a relevant factor not only in terms of connections and central attributes but also in terms of bridge symptoms and comorbidity. This framework extends to the study of symptomatic “comorbidity.”

## Introduction

Clinical and psychopathological research in the child and adolescent population typically tends to group the symptoms of different disorders, dimensions, or spectrums accordingly to classificatory taxonomies such as the *Diagnostic and Statistical Manual of Mental Disorders* and the *Hierarchical Taxonomy of Psychopathology* (HiTOP) [1–4]. The findings of psychopathological research show that many emotional and behavioral problems occur in childhood and adolescence [5, 6]. Based on this empirical research, child psychopathology has been divided into two basic broad-spectrum dimensions or syndromes, described as internalizing and externalizing problems. Internalizing disorders are defined by a group of emotional and behavioral symptoms comprising withdrawal, anxiety, depression, or somatic complaints, whereas externalizing disorders are characterized by disordered symptoms such as rule-breaking and aggressive behavior. Other problems of a mixed and contextual nature (e.g., issues with attention, thinking, and social relationships) are also considered, and “comorbidity” is a term in widespread use to describe the presence of two or more disorders at the same time. In such cases, it is common to discover the presence of depressive disorders alongside anxiety, obsessive–compulsive, conduct, substance use, and eating disorders [7–11]. Comorbidity has become a relevant issue in clinical practice due to its serious impact on the life course and as a major factor in disability or death due to suicide [12].

In recent years, researchers have sought to improve the understanding of psychological problems by proposing new theoretical and methodological paradigms, including network analysis. In this model, disorders and their comorbidity are understood as a dynamic system in which constituents interact, forming a complex network of nodes and connections [13]. It is assumed that there is no single underlying causal relationship between the components [13, 14] and that the dynamic of components generates a particularly stable structure [15]. From a standard pragmatist position, traditional latent variables (e.g., depression or anxiety) models for psychological measurement have proposed that behaviors are perceptible manifestations that provide an operational framework for inferring psychological attributes as well as for identifying a potential common underlying mechanism (a common cause that generates and explains significant correlations of behaviors). Despite their apparent differences, as Epskamp et al. suggest [16], network modeling and latent variable modeling can be considered complementary in methodological terms, as articulated in a combined latent network approach.

In addition, the co-occurrence and the strong relationships between psychological disorders detected in various studies point both to the interactive dynamics of symptoms of discreet disorders and the existence of symptoms shared between disorders [7]. That is to say, two disorders can appear together with bridge symptoms (symptoms that connect both disorders), with the activation of one disorder triggering another, or symptoms may be shared between disorders [17]. These types of symptoms help to explain and understand the comorbidity between diagnoses and to represent theoretical models through measurement.

Previous research with children and adolescents has conducted network analysis on internalizing and externalizing symptoms [18–22]. However, these studies have not been analyzed from a multi-construct or attribute network approach. This perspective operationalizes all the variables included in a network, producing information on an intermediate network level, and provides an insight into groupings of nodes based on their content similarity and grouping interconnections [23]. Symptomatological explorations through network analysis include studies such as those by McElroy et al. [20] and McElroy and Patalay [21]. In a sample of 1,147 children aged from 5 to 14 years, the first study identified four core symptoms that explained the maintenance of the network of mixed anxiety and depression: “anxious,” “sad,” “nervous,” and “worthless.” The second study found that feelings of restlessness, fatigue, and fear were central symptoms of an internalizing symptom network in a clinical sample (*n* = 37, 162) of 8–18 years-olds.

In a longitudinal analysis, Funkhouser et al. [22] found three communities of symptoms categorized through the respective domains of inattention, externalizing problems, and internalizing problems. The core symptoms of these three domains that best predicted the others were depressive mood, inattention, and worry. Regarding externalizing problems, Hukkelberg [19], using a sample of parent-reported data from 551 Norwegian children (aged 3–12) with moderate to high levels of conduct problems (inattention and oppositional defiant behavior) showed that four behaviors were strongly paired: stealing and lying, and physically and verbally fighting with siblings.

Overall, previous research has focused on centrality measures and their connectivity to identify the most dominant symptoms, but to our knowledge, no previous study has been conducted with children to explore the hierarchical arrangement and attribute connections [23]. Thus, the first aim is to analyze the connectivity between different psychological attributes. Regarding this aim, based on previous research findings [11, 19–22], we anticipated finding (hypothesis 1) a pair of attributes with moderate-strong connections such as anxiety–depression as the central attributes of internalizing problems, with rule-breaking behavior as a central attribute of externalizing difficulties. As a second aim, moving beyond the specific centrality aspects of the network, we also estimated the hierarchical arrangement of and the interconnections between psychological attributes based on both the tripartite model [24, 25] and research into general distress predisposition (e.g., trait anxiety, neuroticism) as a common component of negative affectivity [26, 27]. To our knowledge, no previous studies have been conducted on children to explore the hierarchical arrangement and the grouping of nodes. However, it is expected (hypothesis 2) to find a general dimension that represents the core general distress or negative affectivity and secondary dimensions that emerge from the primary core dimension. Finally, the third aim is to explore the symptomatic comorbidity between depression and other internalizing, externalizing, and personal-contextual problems. Given recent findings about the dynamics of psychological pathologies and their comorbidity in children and adolescents, it is expected (hypothesis 3) to find bridge symptoms related to social and interpersonal problems. For instance, feelings of lack of love and loneliness have explained the interconnection or comorbidity between the symptom communities of discrete disorders [11, 19, 28].

## Methods

### Participants

The sample comprised 986 Spanish children and teenagers, with ages ranging from 9 to 18 years (*M* = 13.09, SD = 2.01). The distribution of participants according to age and sex variables was homogeneous (girls = 55%). Participants were selected from fifteen schools from eight different Spanish cities (Cádiz, Algeciras, Jaen, Logroño, Vitoria, Burgos, Segovia, and Madrid) located in five regions that cover the north (La Rioja and Basque Country) south (Andalusia) and the middle of Spain (Madrid and Castilla y León). In line with the Spanish education system, the participants were drawn from three different educational stages (primary: 9–11 years of age, secondary: 12–15, and bachelor’s degree: 16–18). The distribution of the family structures was: (a) father, mother, and children living together (84.9%); (b) mother living with children (6.2%); (c) father with children (1.1%); and (d) shared custody (5.5%).

### Procedure

The study was approved and authorized by the Ethics Commission of the National University of Distance Education (UNED). We requested authorization from the schools and sought informed consent from the participants' parents and the participants themselves. Participation was voluntary. Individual questionnaires were identified by a code to ensure anonymization. Data collection was conducted in classrooms in school buildings under researcher supervision for two 45 min periods adjacent to recess.

### Measures

Clinical symptoms of depression were measured using the *Center for Epidemiological Studies Depression Scale for Children and Adolescents*, CES-DC [29–32]. This scale consists of 20 items with four Likert-type response options (1 = *almost nothing* to 4 = *a lot*). It measures four dimensions or subscales and we estimated their reliability using the Omega coefficient (ꞷ) [33, 34]. The attributes are: depressed affect (*ꞷ* = 0.91), somatic problems (*ꞷ* = 0.73), interpersonal problems (*ꞷ* = 0.72), and positive affect (*ꞷ* = 0.75). All these dimensions were included in the depressive symptom community, except positive affect, which was analyzed as an independent attribute due to the contrary direction of this measure compared with the other dimensions of depression.

The Youth Self-Report (YSR) [35, 36] is a self-report questionnaire that evaluates emotional and behavioral problems in children and adolescents. It has 112 items measured on 3 Likert-type scale response options (0 = *not true* to 3 = *true, very often, or fairly often*). The attributes are: *internalizing problems,* such as anxiety–depression (*ω* = 0.79), anxiety (*ω* = 0.66), withdrawn (*ω* = 0.70), and somatic complaints (*ω* = 0. 71); *externalizing problems,* such as aggressive behavior (*ω* = 0.84) and rule-breaking behavior (*ω* = 0.79); *personal-contextual problems,* such as thought problems (ω = 0.75), social problems (*ω* = 0.71), attention problems (*ω* = 0.78), and other problems (*ω* = 0.51).

### Data analysis plan

The R program version 4.0.3 [37] was used to conduct all analyses. First, we performed an exploratory descriptive analysis of the items. The highest percentage of missing data was 2% for CES-DC items and 3% for YSR items. Taking into account the standard practice of imputing when the quantity of missing data is less than 20% [38], we treated missing data with multiple imputations, recommended as the best method for Likert-type scales [39, 40], even for CES-DC [41] using the random forest technique [42]. We conducted it with the MICE package, version 3.10.0 [43].

We analyzed the structure and dynamics between the psychopathological attributes by estimating a network of regularized partial correlations that shows the most statistically significant relationships and in whose calculation a regularization technique is applied (*EBICglasso* method) [16, 44–47]. The dynamics and relevance of attributes were evaluated using standard centrality measures: strength (St), betweenness (Bet), closeness (Clo), and expected influence (EI) [44, 45].

To study the hierarchical organization of the attribute network, we estimated a minimum spanning tree (MST). With this analysis, central nodes were identified based on local connections [23]. We also evaluated the interconnections between attributes considering the structure of the community by estimating the participation coefficient (PC) and participation ratio (PR). For PC, values closer to zero suggest greater connectivity within the community and values closer to one suggest greater connectivity between communities. For PR, nodes with higher values have a greater total force and a larger number of connections [23, 48].

Subsequently, to explore the symptomatic comorbidity between depression and internalizing, externalizing and personal-contextual problems we calculated the comorbidity centrality measures *bridge strength, bridge closeness, bridge betweenness,* and *bridge expected influence step 1 and step 2*. For each bridge centrality measure, we identified *bridge symptoms*, considered as those with 20% of their nodes with the highest score, located above the 80th percentile, which was set as the cut-off point where an acceptable balance between sensitivity and specificity occurs [17]. In this analysis, communities of symptoms are defined as theoretical attributes or psychopathological disorders, so the nodes grouping belong in a pre-existing way.

The regularized partial correlation networks and standard centrality measures were estimated with the *qgraph* and *centralityPlot* functions in the *qgraph* package, version 1.9.2 [49]. The *MST* was calculated using the *igraph* package, version 1.2.5 [50], the *PC* with the *NetworkToolbox* package version 1.4.1 [51], and the *PR* with *brainGraph* package, version 3.0.0 [52]. The bridge centrality measures were conducted with the *bridge* function in the *networktools* package, version 1.2.3 [53]. Lastly, a co-occurrence graph was used to visualize the bridge symptoms [54].

## Results

### Attributes network: centrality and connectivity

**Fig. 1:**
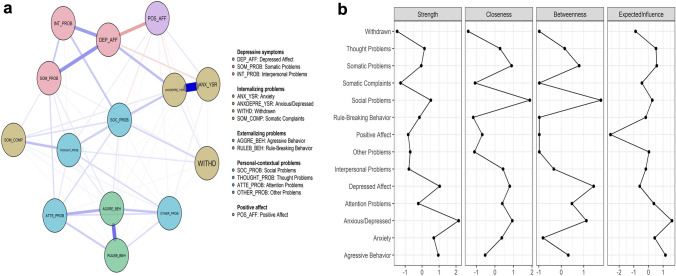
Regularized partial correlation attributes network (Panel **a**) and standard centrality measures graph (Panel **b**) of depression and other personal-contextual problems in children and adolescents

To analyze the connectivity between the attributes (see Fig. 1), a regularized partial correlation network and a centrality measures graph are presented. In Panel a, the prominent position of social problems and anxiety–depression is shown. In addition, strong relationships are identified between depressive affect and the other dimensions of depression, such as interpersonal problems, somatic symptoms, and positive affect. This last attribute stands out through its strong inverse relationship with depressive affect.

In Panel b, the attributes that best activate the network in terms of the number of connections and their weights are anxiety–depression (St = 2.15, EI = 1.60), depressive affect (St = 1.03), and aggressive behavior (St = 0.95, EI = 1.15). Moreover, the best intermediaries are anxiety–depression (Bet = 1.12), depressive affect (Bet = 1.44), and social problems (Bet = 1.76), which activate the network in the shortest time (Clo = 1.88).

**Fig. 2:**
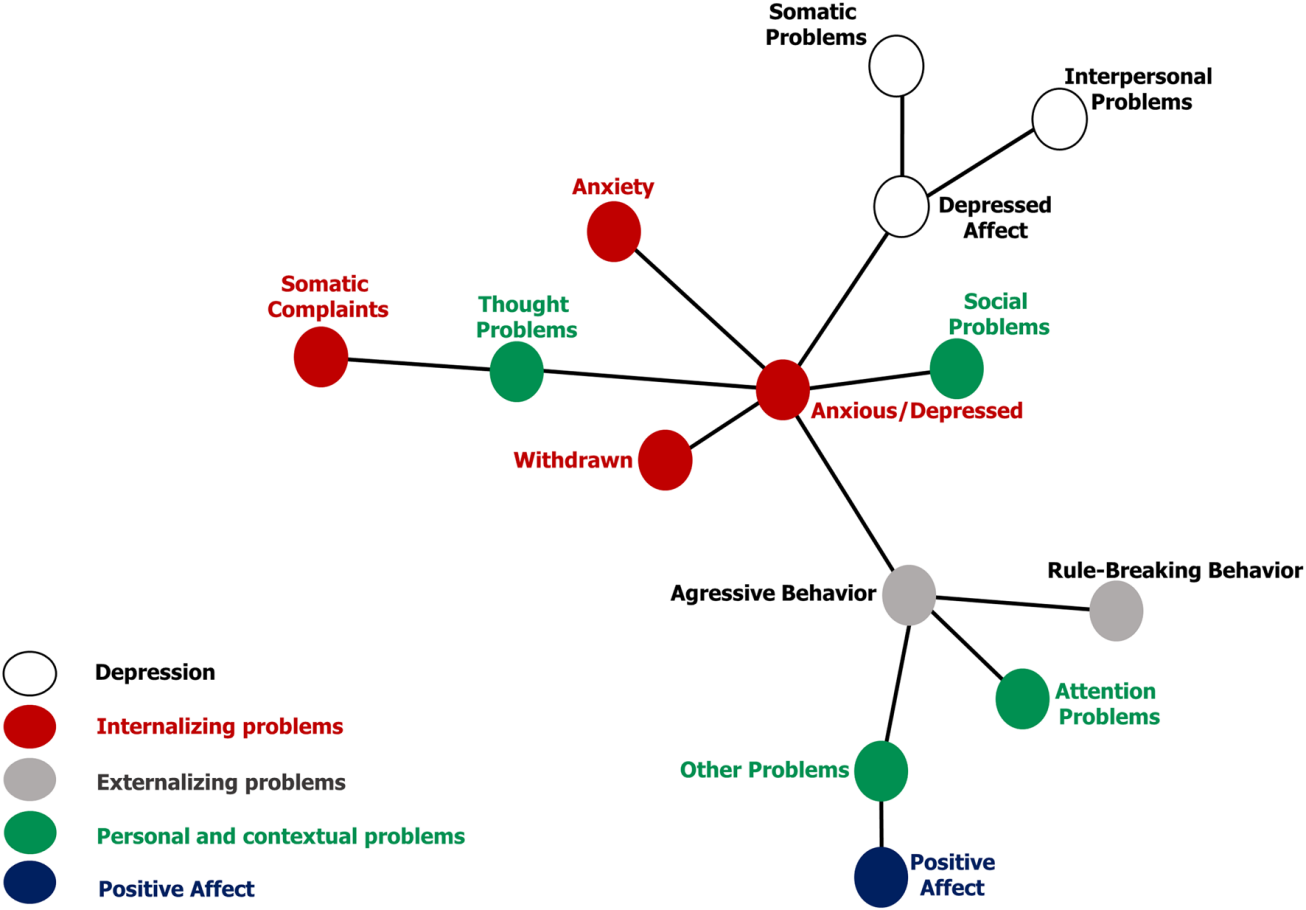
Minimum spanning tree (MST) of depression and other personal-contextual problems in children and adolescents

The MST presented in Fig. 2 shows the hierarchical dynamics between attributes. Mixed anxiety–depression was identified as the core and the other attributes were hierarchically connected to it by being positioned in six branches that are differentiated according to their theoretical contents. The attributes of clinical depression were grouped into one branch: depressive affect, somatic symptoms, and interpersonal problems. This shows the relationship between anxiety–depression (central dimension) and depressive affect. Then, behaviors of contextual, inter-relational, and intrapersonal nature are allocated to other branches, with social problems in the second branch, anxiety in the third branch, problems with perceptual-somatic content, such as thinking problems and somatic complaints, in the fourth branch, and withdrawal behaviors in the fifth branch. Lastly, the sixth branch groups attributes of externalizing manifestation, such as opposition/disinhibition (e.g., aggressive behavior that is strongly linked to rule-breaking and attention problems), other problems (e.g., poor diet, overweight, nail-biting, excessive sleeping), and positive affect, which relates to the perception of pleasant feelings and positive expectations of social relationships and life.

**Fig. 3:**
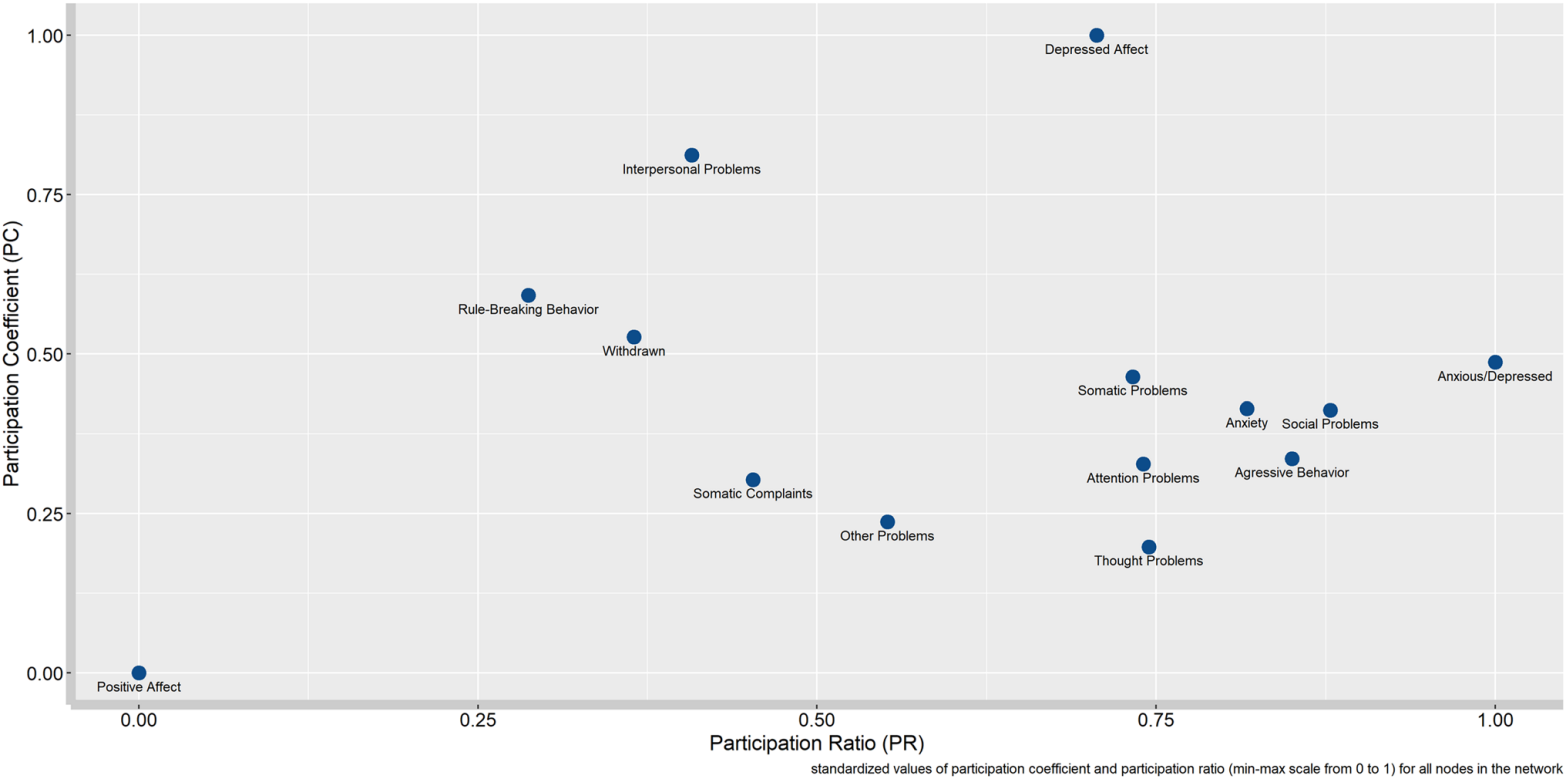
Scatterplot of the standardized values of the participation coefficient (PC) and participation ratio (PR) of depression and other personal-contextual problems in children and adolescents

In Fig. 3 the standardized values of the participation ratio (PR) and the participation coefficient (PC) are shown to represent the distribution and weight of the connections between attributes. Although the dimensions of anxiety–depression, social problems, aggressive behavior, and anxiety have the greatest strength and number of connections (PR > 0.75), they are moderately distributed throughout the network (PC < 0.50). This indicates that their most central connections are established with the attributes of their own community or theoretical groups: anxiety–depression with internalized problems, social problems with personal-contextual problems, and aggressive behavior with externalized problems. In sum, they have more influence within their community.

Alternatively, it can be observed according to the distribution of connections that depressive affect and interpersonal problems are the nodes with the highest participation coefficients (PC > 0.75), which means their connections are evenly distributed with the attributes of other communities or theoretical groups. In the case of positive affect, it appears as a node with the lowest PR and PC values, indicating that it has fewer and weaker connections with other psychological attributes.

### Symptomatic comorbidity between depression and other psychological problems

**Fig. 4:**
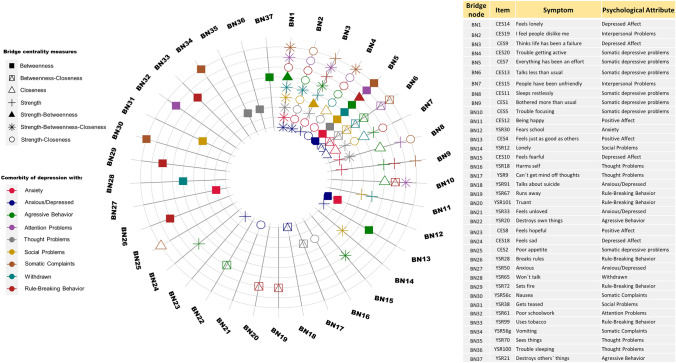
Co-occurrence graph of bridge symptoms of symptomatic comorbidity between depression and other personal-contextual problems in children and adolescent

In Fig. 4, we present the 37 bridge symptoms identified in all networks. In the co-occurrence graph, each circular line represents the depression comorbidity network with a specific construct (distinguished by colors) and the diagonal lines correspond to the identified bridge symptoms. The icons (listed in the legend in the upper left corner) represent the bridge centrality measure with which that symptom was identified.

Considering each bridge centrality measure and their combinations, the most relevant symptoms were “feels lonely” (CES14), “I feel people dislike me” (CES19), “perceive life has been a failure” (CES9), and “trouble getting active” (CES20). Notably, “everything has been an effort” (CES7) would be the most common intermediary among all comorbidity networks. Likewise, if we observe “bridge intermediation-bridge closeness”, the somatic depressive symptom “talks less than usual” (CES13) shares both properties in the comorbidity networks between the depressive symptoms and other disorders, except for rule-breaking.

The symptom “bothered more than usual” (CES1) appears as a bridge symptom with strong connectivity in the networks with anxiety, rule-breaking behavior, aggressive behavior, and somatic complaints. Perceiving that “people have been unfriendly” (CES15), as a depressive symptom is also related to the dimension of interpersonal problems, being a bridge symptom with high prevalence in its comorbidity with thought problems and aggressive behavior; high closeness for anxiety, anxiety–depression and withdrawn comorbidity, and both strength-closeness in social problems and rule-breaking.

Four bridge symptoms occur among different psychological problems: loneliness (CES14-YSR12), suicidal thinking-self-harm (YSR91-YSR18), talking less (CES13-YSR65), and sleep problems (CES11-YSR100) as well as two symptoms related to the academic context such as “fears school” (YSR30) from the anxiety dimension and “poor schoolwork” (YSR61) from the attention problem dimension.

In addition, there were bridge symptoms identified only in comorbidity networks between depression and each specific psychological problem: (a) in the aggressive behavior network, “feels just as good as others” (CES4), which belongs to positive affect, as well as “feels fearful” (CES10) and “feels sad” (CES18) for depressive affect; (b) in social problems, “gets teased” (YSR38) and “lonely” (YSR12); (c) in somatic complaints, symptoms referred to eating behavior and gastrointestinal problems such as “poor appetite” (CES2), “nausea” (YSR56c), “vomiting” (YSR56g), and lastly, (d) “breaks rules” (YSR28), “uses tobacco” (YSR99), “runs away” (YSR67), “truant” (YSR101), and “sets fire” (YSR72) for rule-breaking.

## Discussion

The general aim of the present study was to explore the dynamic and symptomatic comorbidity of the psychological attributes of depression with other internalizing, externalizing, and personal-contextual problems in children and adolescents. The first specific aim was to analyze the connectivity among different psychological attributes. Our findings evidence the significant role of mixed anxiety–depression as an activator and intermediary of psychopathologies, which is consistent with studies where anxiety is a relevant comorbid depressive disorder [55, 56]. As we expected in hypothesis 1, anxiety–depression was supported as a central attribute of internalizing problems.

Evidence of connections and comorbid dynamics between depression and anxiety has been reflected in different theoretical models. Particular models (e.g., the tripartite model) propose that both conditions are constructs or phenomena with particular characteristics: for some, depression is characterized by low positive affectivity and high sadness or negative affect, while anxiety is characterized mainly by high levels of positive affect, physiological hyperarousal, and fear [24, 25].

Regarding hypothesis 1 we did not find, as expected, that rule-breaking behavior was a central attribute of externalizing problems. However, aggressive behavior was one of the constructs that most intensively activate the network in terms of the number of connections and their weight. Although aggression is a behavior included in the same broad externalizing dimension as rule-breaking, it tends to be less disruptive and more socially accepted. The absence of children at social high risk or social exclusion in the sample for this study could be a factor in this result.

In addition to anxiety–depression and aggressive behavior, the dynamics of symptom interrelations showed an unexpected result: the manifestation of social problems was also distinguished as a relevant factor. This is discussed below in relation to the comorbidity symptoms, the area pertaining to the third specific aim of our study.

A second specific aim was to estimate the hierarchical arrangement and the interconnections between the psychological attributes based on the tripartite model [24, 25]. Hypothesis 2 was supported by consistent findings for anxiety–depression as a central attribute with moderate-strong connections: a general dimension as a general distress or negative affectivity emerges from the primary core dimensions. Evidence from applied clinical settings suggest beyond their comorbidity anxiety–depression could emerge as a separate phenomenon, termed “distress”, which is characterized by a generalized negative affect [24].

Anxiety–depression as a central attribute is also consistent with a neurotic personality trait as a dimension of vulnerability that increases the risk of psychological problems [26, 27, 57]. In addition, we observed a strong inverse relationship between depressive affect and positive affect in the regularized network. This finding supports a theoretical model in which both positive and negative affect are considered essential but independent dimensions of depression [56], defined as a mixed state of high negative affect (shared with anxiety problems) and specifically low positive affect [24, 25, 28].

The third aim was to explore the symptomatic comorbidity between depression and other internalizing, externalizing, and personal-contextual problems. As mentioned above, the manifestation of social problems was also distinguished as a relevant issue, not only in terms of connections and central attributes but also in terms of bridge symptoms and comorbidity, as we expected in hypothesis 3. Social problems are highlighted by their role as a strong intermediary between other problems as well as their strong connections with internalized problems. This supports the relevance of social competence for children and adolescents detected in other research [11, 58–60]. This finding acquires relevance during adolescence, given the influence of peers and groups in the search for identity and the improvement of various intrapersonal capacities. Peer rejection, isolation, lack of social skills, and other interpersonal difficulties have been associated with the presence of depressive disorders [55, 60]. As for aggressive behavior, despite our agreement that it should be considered a distinct phenomenon beyond other psychological problems such as depression and anxiety, our findings also support the conclusion that children who initially present with depressive-anxious symptoms may also show various aggressive behaviors linked, in part, to a low tolerance for frustration or a high vulnerability triggered by life events during their development towards adulthood [11, 61–63].

In addition to confirmation of our hypotheses in relation to the dynamics of psychological problems and their comorbidity in children and adolescents, this study offers other important insights. Firstly, strong associations between attributes of the same nosology support the theoretical proposals of dimensional taxonomies for clinical diagnosis focusing on symptoms grouped into “spectra” [1, 2, 4]. Secondly, the emerging role of psychopathologies is also sustained by the findings in relation to symptomatological comorbidity, where symptoms of both depressive affect and somatic nature are highlighted. Somatic symptoms were related to anhedonia, inactivity, sleep problems, fatigue-tiredness, and gastrointestinal issues. This was in accordance with findings in adolescents which highlight anhedonia as characterized by losing a sense of connection and belonging as well as questioning the sense of self, purpose, and the bigger picture [64]. This is demonstrated in our study through the manifestation of “thinks life has been a failure” as one of the strongest bridge symptoms. On the same track, studies of sleep quality demonstrate the association between feelings of fatigue and physical activity frequency, whereby fatigue symptoms improve as physical activity increases among those with poor sleep quality [65]. As for gastrointestinal issues, studies have clearly identified the activation of physiological vegetative manifestations as symptoms of depression [9]. Thirdly, results related to the comorbidity of depressive symptoms with externalizing problems and anxiety should be considered from a developmental perspective. This is the case for the attribute “bothered more than usual” which is identified with the characteristic adolescent tendency to become easily irritated [66]. Behaviors that express opposition to authority and the search for autonomy or reaffirmation of identity, such as “destroys own and other´s things”, can also be an expression of emotional distress and can arise at the same time as a response to it [67, 68]. On the other hand, there can also be inverse dynamics where violent behavior generates rejection in others, isolation, or performance difficulties, which can lead to depression [61, 69, 70]. This indicates the way reciprocal mechanisms operate in comorbidity. Fourthly, other bridge symptoms with anxiety are clearly related to the attributes “fears school” and “poor schoolwork” relating to academic contexts. The first could be a potential indicator of school phobia or even bullying; the second is an attribute associated with low self-efficacy and poor self-esteem derived from academic problems; both are connected with anxiety and externalized problems [61, 71, 72]. Finally, our findings concerning depressive symptoms reveal them to be markedly intrapersonal in nature, rather than purely interpersonal experiences. These results support existing research into ontological relationships with cognitive, motivational, or perceptual phenomena such as cognitive biases and irrational beliefs alongside the appearance and maintenance of different mental disorders [73, 74] or the comorbidity between depressive and obsessive–compulsive behaviors [8, 10, 17, 75]. In general, bridge symptoms of intrapersonal perception (e.g., “feels lonely”, “I feel people dislike me”, “people have been unfriendly”) which reflect the distance between the desire to establish social relationships and the reality of such relationships—as well as the presence of devaluation or ruminant thoughts about social identity—have been shown to be consistent features of comorbidity in depression [69, 76]. Lastly, as we detected in comorbidities with thought problems and mixed anxiety–depression, the symptoms of hopelessness and suicidal ideation (such as “harms self” and “about suicide”) are consistent with the depression model of helplessness or hopelessness as an inducer of suicide and self-injurious behaviors [58].

## Conclusions

Taking all these results as a whole, three main conclusions can be made: (1) Three central attributes appear with moderate-strong connections: anxiety–depression as the central attributes of internalizing problems; aggression behavior as the central attribute of externalizing problems, and social problems as the central attribute of personal-contextual issues; (2) According to the tripartite model, a general predisposition to distress derives from an anxiety–depression dimension that represents the core negative affectivity that may explain comorbidity between syndromes in children; (3) Comorbidity between depression and other internalizing, externalizing, and personal-contextual problems shows that the manifestation of social problems is distinguished as a relevant issue in terms of bridge symptoms. However, the dynamics of psychological problems and their comorbidity in children and adolescents involve somatic symptoms, and depression is linked with the perception of interpersonal relations and anxiety with academic and school contexts. Finally, a developmental manifestation and reciprocal mechanisms among symptoms should be considered in the comorbidity of psychological disorders in children and adolescents.

In sum, the results highlight conceptual, methodological, and practical implications including the suggestion that bridge symptoms could be used in the detection of children at risk of developing psychological disorders. Attention could focus on identifying these symptoms in everyday situations at home and in educational contexts. Professional psychologists working in different areas of health, education, and community support could address the presence of these behaviors in the evaluation of the evolution or prognosis of psychological problems. The identified behaviors could serve as elements for the construction of new models of comorbidity and as essential risk components in the design of preventive actions.

## Limitations

Despite the results obtained, this work has limitations. Among the symptoms studied, those with a more physiological basis (e.g., tachycardia, dizziness, or shortness of breath) have not been considered. The analysis was undertaken with cross-sectional data, so it was not possible to study dynamic sequences. Finally, the participants were selected from a broad, non-clinical population, so a categorical clinical diagnosis of anxiety or depression was not possible. Another further limitation is related to this specific sample, meaning that these results cannot be generalized to broader populations. Taking these limitations into account, we suggest future research could aim to study psychopathologies using both attribute and symptomatic approaches to capture symptom fluctuations, specific relationships between psychological attributes, and other factors that vary over time.

## Data Availability

Not available.
